# On the Temporal Characteristics of Performance Variability in Attention Deficit Hyperactivity Disorder (ADHD)

**DOI:** 10.1371/journal.pone.0069674

**Published:** 2013-10-02

**Authors:** Bernd Feige, Monica Biscaldi, Christopher W. N. Saville, Christian Kluckert, Stephan Bender, Ulrich Ebner-Priemer, Klaus Hennighausen, Reinhold Rauh, Christian Fleischhaker, Christoph Klein

**Affiliations:** 1 Department Psychiatry and Psychotherapy, University of Freiburg, Freiburg, Germany; 2 Department of Child and Adolescent Psychiatry and Psychotherapy, University of Freiburg, Freiburg, Germany; 3 School of Psychology, Bangor University, Bangor, United Kingdom; 4 Department of Child and Adolescent Psychiatry, University of Dresden, Dresden, Germany; 5 Department of Applied Psychology, Karlsruhe Institute of Technology, Karlsruhe, Germany; Centre national de la recherche scientifique, France

## Abstract

Increased intra-subject variability of reaction times (ISV-RT) is one of the most consistent findings in attention-deficit/hyperactivity disorder (ADHD). Although the nature of this phenomenon is still unclear, it has been hypothesised to reflect interference from the Default Mode Network (DMN). So far, ISV-RT has been operationally defined either as a frequency spectrum of the underlying RT time series, or as a measure of dispersion of the RT scores distribution. Here, we use a novel RT analysis framework to link these hitherto unconnected facets of ISV-RT by determining the sensitivity of different measures of RT dispersion to the frequency content of the underlying RT time series. N=27 patients with ADHD and N=26 healthy controls performed several visual N-back tasks. Different measures of RT dispersion were repeatedly modelled after individual frequency bands of the underlying RT time series had been either extracted or suppressed using frequency-domain filtering. We found that the intra-subject standard deviation of RT preserves the “1/f noise” characteristic typical of human RT data. Furthermore and most importantly, we found that the ex-Gaussian parameter τ is rather exclusively sensitive to frequencies below 0.025 Hz in the underlying RT time series and that the particularly slow RTs, which nourish τ, occur regularly as part of an quasi-periodic, ultra-slow RT fluctuation. Overall, our results are compatible with the idea that ISV-RT is modulated by an endogenous, slowly fluctuating process that may reflect DMN interference.

## Introduction

### 1: Intra-subject variability of reaction times is a current topic in ADHD research

Increased intra-subject variability of reaction times (ISV-RT) in attention-deficit hyperactivity disorder (ADHD) has turned from a neglected abnormality [1-3] to a field of productive research [4-9]. This increase in interest is in part due to evidence that ISV reflects a stable trait [10-12] and possibly a unitary construct that generalises across a broad range of tasks and sensory modalities [7,13,14]. Furthermore, ISV is both familial [15,16] and hereditary [17,18], thus qualifying as a candidate endophenotype of ADHD [1,19].

### 2: Specific measures of RT dispersion are required to characterise increased ISV in ADHD

However, while it is clear that patients with ADHD show increased RT variability, it is not clear what this actually reflects. Part of this ambiguity stems from the fact that in the vast majority of ADHD studies ISV has been quantified through the intra-subject standard deviation of RT (RTSD [8]). By summing up all deviations from the average, RTSD becomes sensitive to all such sources of ISV (e.g., fluctuations on different frequency scales, irregularly occurring particularly fast or slow responses, (non-)linear trends etc.), and is thus specific to none. Furthermore, while RTSD assumes Gaussian normality of the RT distribution, RT distributions are in fact skewed, with a long tail of slow responses. RT distributions instead resemble ex-Gaussian distributions, that is, convolutions of a Gaussian distribution with an exponential distribution [20-22]. The ex-Gaussian distribution parameters µ and σ represent mean and standard deviation, respectively, of the Gaussian component, while τ represents mean and standard deviation of the exponential component. The increased mean RT and RTSD found in ADHD appear to reflect increased τ, with normal µ and σ [23], suggesting that increased ISV could be the consequence of increased density of slow responses. Slow responses have been found to be preceded by reduced pre-stimulus activity in fMRI studies with healthy participants [24]; they may thus reflect temporary reductions in preparatory attention and, with regard to ADHD, have been metaphorically considered by some authors as “lapses in attention” due to a defective effort control mechanism [23]. Increased τ has been replicated consistently for children, adolescents and adults with ADHD so far [25-28]. But abnormally high σ has also been found in most of these studies (with the exception of the adults in [27]), suggesting that whatever drives the slow responses reflected in τ is not the only “ingredient” of increased ISV in ADHD.

### 3: Investigating temporal structures of RT time series reveals important features of ISV

While measures such as τ identify increased ISV as an abnormality in the RT *distribution*, other approaches consider the same data as reaction *time series* [29,30] and search for abnormalities in patients in the temporal structure of the time series. Castellanos et al. [3] were the first to analyse the periodic structure of RT series in patients with ADHD and reported increased power of RT oscillations around 0.05Hz, thus pointing to fluctuations with a cycle length of about 20 seconds. This finding has been replicated conceptually in other studies [11,31,26,32].

### 4: The aims of the present study

**Figure 1:**
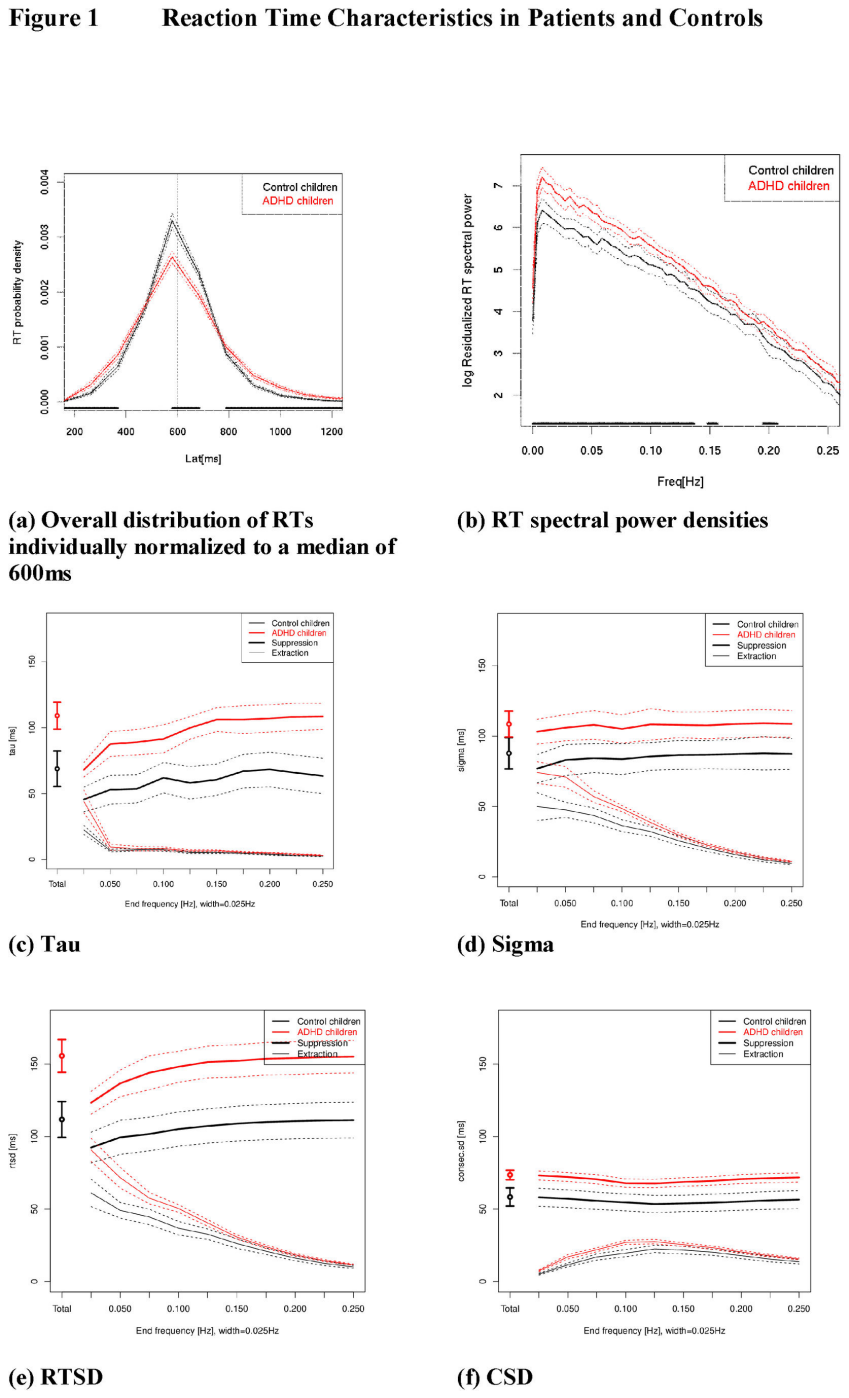
Averages (solid lines) with 95% confidence intervals (dotted lines) are shown for controls (in black) and patients (in red). The black bar above the x-axes in figures a and b indicates an uncorrected point-wise “p<.05” difference between the groups to provide an indication where group effect sizes are considerable. “Suppression filters” (upper, thick lines in Figure 1 c-f) demonstrate the impact of removing each particular frequency band from the RT time series on the group aggregate of the individual variability scores; the “extraction filters” (lower, thin lines in Figure 1 c-f) show the said measures of variability for each RT time series frequency band alone. Suppression and extraction filters are therefore complementary ways of visualising one and the same relationship.

Referring to the first four sections of this article, the aims of the present study are as follows. As the same RT data can be considered as a dispersion of RT scores (see section 1.2) or a temporally structured RT time series (section 1.3), the aim of the present study is the characterisation of commonly used RT dispersion measures in terms of their frequency specificity. To that end, we focused on RTSD as the “gold standard” measure of ISV in ADHD, on τ and σ as the two ISV components of the ex-Gaussian model that contribute to RSTD, and on the consecutive standard deviation (“CSD”) as the standard deviation of the differenced time series (i.e., difference between subsequent RTs [33]) that has been considered to quantify moment-to-moment fluctuations of the process under scrutiny [7,34,35]. Our characterisation of dispersion measures by their frequency specificity employs standard filters to extract or suppress certain frequency bands in the RT time series before re-modelling the measures of RT dispersion (see methods and Figure 1).

## Materials and Methods

### 1: Ethics statement

Our study had been approved by the ethics committee of the Universitätsklinikum Freiburg. Written informed consent was obtained from all parents whose child participated in the study.

### 2: Participants

Outpatients of the Department of Child and Adolescent Psychiatry who had received a diagnosis of ADHD according to DSM-IV criteria were recruited for the study. Diagnoses had been routinely obtained by experienced clinical psychologists and psychiatrists on the basis of (a) Conner’s parent and teacher rating scales, (b) medical files of the treating psychiatrist, and (c) interviews with the child and accompanying parent about the child’s patient history. Diagnostic information was complemented (d) by a video-taped clinical observation of child behaviour obtained in a group session with standardized situations (e.g., structured play, homework, test taking). An ADHD diagnosis was given if these sources yielded converging information. The diagnostic interview K-SADS-PL [36] was administered to parents and patients separately to confirm the ADHD diagnosis, accomplish DSM-IV-based subtype classification and screen for co-morbid disorders. Parents filled in the Child Behaviour Check List (CBCL [37]). Data from N=27 patients with an ADHD diagnosis (all males, mean age ± SD: 11.1±0.9 years, range: 9.9-12.5, mean IQ ± SD: 98.0±11.0, range: 81.6-116.9) were available for statistical analyses. Eight patients were of combined type (DSM-IV code: 314.01), 9 predominantly inattentive (314.00), 3 predominantly hyperactive-impulsive (314.02), and 6 were not further specified according to the K-SADS-PL algorithm (that is, “NOS”). At the time of the study, 21 patients were taking methylphenidate medication, which was interrupted for more than 24 hours before the testing day. In some participants ADHD was accompanied by one or more co-morbid disorders, namely conduct disorder (N=3) and/or encopresis, enuresis (N=3). N=26 healthy controls (all males, mean age ± SD: 11.7±0.9 years, range 10.0-12.0; mean IQ ± SD: 99.6±14.3, range 85.1-130.8) were recruited through advertisements in schools or local newspapers and had no history of psychiatric or neurological disorders, as reported by the parents upon telephone interviewing. Patients and controls differed significantly in most CBCL subscales (see Table **1**), but not in IQ, as assessed with Raven’s Standard Progressive Matrices, a well-established proxy of general intelligence (F_1,51_<1, p=0.64; Raven’s SPM [38]).

**Table 1:** Means (±SD) of CBCL/4-18 Subscales and CBCL/4-18 Total Score.

	**TD**	**ADHD^a^**	**z**	***p***
Withdrawn	1.50 ± 1.50	2.50 ± 2.36	1.49	.14
Somatic Complaints	0.69 ± 0.79	1.63 ± 1.84	1.90	.06
Anxious/Depressed	1.73 ± 2.32	4.79 ± 4.42	2.97	<.003
Social Problems	0.77 ± 1.03	3.46 ± 2.59	4.41	<.0001
Thought Problems	0.31 ± 0.62	1.13 ± 2.07	1.61	.11
Attention Problems	2.19 ± 2.33	6.21 ± 3.60	4.12	<.0001
Delinquent Rule-Breaking Behaviour	1.12 ± 1.51	3.46 ± 3.02	3.23	.002
Aggressive Behaviour	4.77 ± 4.25	13.50 ± 9.03	3.97	<.0001
Internalizing	3.77 ± 3.57	8.50 ± 7.30	2.65	.009
Externalizing	5.88 ± 5.35	16.96 ± 11.75	3.81	.0002
Total Problems	14.65 ± 11.51	39.58 ± 26.44	3.99	<.0001

Note. ^a^N=24 because (i) two ADHD patients did not return questionnaires, and (ii) one had too many missing values; TD = Typical Development, ADHD = Attention-Deficit/Hyperactivity Disorder; z = z approximation of the Wilcoxon-Mann-Whitney test;

### 3: Hardware and Procedure

Data were collected as part of a larger study that also included EEG recordings (Feige et al., in preparation). Stimuli were presented on a 17" VGA monitor with "Presentation" (Neurobehavioral Systems, Albany, CA) and responses were collected with a self-made response pad consisting of two separate push-buttons of 3x3 cm size each. Given that increased ISV in ADHD may be influenced by the task context in which it is observed, we experimentally manipulated two well-replicated factors of cognitive deficits in ADHD, working memory [39] and temporal processing [40], in a letter version of the N-back task. During *0-back tasks*, presentation of an “E” was defined as an “event”, presentation of all other letters as “non-events”. During *1-back tasks*, the stimulus types event vs. non-event were defined as presentation of a letter that was identical or non-identical with the previous letter, respectively. Event-rate was 25% in all tasks, and participants had to respond as quickly and accurately as possible with the left- or right-hand response buttons to all events and all non-events. The assignment of response hand to trial type was counter-balanced across participants. Stimulus presentation duration was 0.5s. Stimulus-onset asynchrony was 2.5s on average, being fixed in "non-jittered" and varying (2.0-3.0s) in "jittered" conditions. The four task groups (0-back/1-back x jittered/ non-jittered) consisting of 2x180 trials each (with pauses between the two blocks and the four task groups) were preceded by at least 20 practice trials and presented in permutated order across participants of both groups.

### 4: Data analysis

As part of our *exploratory data analysis*, we derived the RT probability density function (showing the relative frequency of responses in 20 ms bins from 0 to 2000 ms; see Figure **1a**) separately for each subject and condition after normalizing the median reaction time to 600 ms by dividing each RT by the median for this subject and condition and multiplying by 600 ms. This processing step was accomplished to visualize group differences in RT distribution. The normalized probability density functions were then plotted with group statistics (mean and 95% CI for each RT bin).

The *Primary data analysis* consisted of three steps. *First*, we differentiated three sources of RT variability: ISV proper, linear trends (e.g., due to fatigue) and event/non-event RT differences (for a taxonomy of sources of ISV, see 41,30). To analyse ISV proper independently of the other sources, we “residualized” RTs by subtracting the fit to a linear model from all task blocks. The linear model included a trial time covariate for linear trends and the factors STIMULUS TYPE (event/non-event) and inter-run factors TASK (0-back/1-back) and JITTER (jitter/no-jitter). The range of valid RT was 200-1500 ms, excluding anticipations or excessively slow responses. This residualisation procedure thus removed all components of intra-subject variability due to task, stimulus manipulations or trends.

*Second*, we derived different measures of dispersion. The intra-individual RT standard deviation (RTSD) was computed for reasons of comparison, being the "gold standard" in the ADHD literature [7]. The "consecutive standard deviation" (CSD [7]) was computed as standard deviation of trial-to-trial RT differences (sqrt(∑(RT_i_-RT_i+1_)^2^/(n-1)); i=trial number, n=number of trials, sqrt=square root). Ex-Gaussian σ and τ were derived using the GAMLSS package for the open-source statistical package R [21,22,42]. Non-residualized measures of central tendency were arithmetic mean (MRT), median (MnRT) and ex-Gaussian **µ**. Error measures were proportion of omission errors (om) and proportion of correct responses (cor).

*Third*, after re-sampling the residualized RT time series to a frequency of 1 Hz by linear interpolation, Fast Fourier Transforms (FFT) were run separately for each subject in each experimental condition to examine the RT variability frequency range (0-0.25 Hz) in bins of 0.004 Hz for both groups.

To determine contributions of fluctuations on different time scales to RTSD, CSD, σ and τ, the same frequency range was subdivided into 10 frequency bands of 0.025 Hz width. For each participant and frequency band separately, RT series of four experimental conditions were filtered with a *suppression filter* (filtering out the given band) and an *extraction filter* (filtering out all bands except the given band), after which the individual RTSD, CSD, σ and τ scores were re-calculated and statistically analysed. The extraction filter thus removes all frequencies except the given band, whereas the suppression filter removes only the given frequency band. Filtering was performed on each residualized RT time series after resampling to 1Hz using linear interpolation. For manipulations in the frequency domain, it is important that the residualisation described above de-means and detrends the RT time series, which effectively suppresses the effects of very slow variations (trends such as effects of fatigue as well as oscillations with a cycle length comparable to the block length or longer, i.e. 0.001 Hz with the block length of 900 s). The resampled time series was zero-padded to the next power-of-two length, converted into the frequency domain using a raw complex-valued FFT, multiplied by the desired frequency characteristic and converted back by inverse FFT. The frequency characteristics were constructed as simple pass- or stop bands with a linear flank of width 0.001 Hz at either side, limiting the steepness of the filter.

As mentioned in the introduction, τ can be viewed quite specifically in terms of particularly slow reaction times interspersed in a background of, on average, faster reactions. Furthermore, the low-frequency components of RT time series should represent relatively long alternating phases of faster and slower reaction times. Therefore, and in order to express both τ and the low-frequency components in the time domain, we carried out the following analysis steps. Particularly slow or fast RTs (residualized, as explained before) within a given block of trials were defined by the theoretical 1% threshold of a Gaussian distribution (median ± 2.33 SD, where SD was computed non-parametrically as IQR/1.349, IQR=Inter-Quartile Range). Next, a running median RT was computed across all other RTs, using a 40 s time window. This "background RT fluctuation" was, in turn, divided into three classes, depending upon whether the current value fell into the lower, middle or upper tercile of all values. Finally, in order to determine whether the particularly slow or fast RTs were associated with the phase of background RT fluctuation, we compared the number of slow or fast RTs within each of the background RT terciles. As a control, we also checked whether the occurrence of omission errors was associated with either class of RT. Separately for slow and fast RTs as well as omissions, the counts for lower, middle or upper RT classes were compared using χ^2^ tests.

### 5: Statistics

Measures of central tendency, dispersion and error were statistically analysed with univariate repeated measures ANCOVA, including the aforementioned with-subject factors NBACK, JITTER and STIMULUS TYPE, the between-subject factor GROUP (patients/controls); age in months was added as covariate to reduce (age-related) error variance (within-group sums of squares).

## Results

### 1: Overall characterisation of group differences and experimental effects

Figure **1a** shows probability density distributions of RTs individually normalized to a median of 600 ms for both groups. Probability density is shown as the relative frequency of responses in 20 ms bins from 0 to 2000 ms. Overall, the patients’ RT distribution appears broader and flatter than that of controls, with higher RT probability densities at both extremes and lower densities around the median due to total probability being fixed to 1. When considering the same RT data as time series, patients show greater spectral power across the entire frequency spectrum, with group differences being particularly pronounced at slow frequencies (<0.1 Hz). The range above .15 Hz shows smallest group differences (see Figure **1b**). Furthermore, while the number of early responses (RT<200 ms) was greater in patients as compared to controls (see Table **2**), the number of particularly slow responses (>1,500 ms) was only somewhat greater in those with ADHD (7.5±14.2) than in healthy children (1.9±3.7; GROUP: F_1,51_=3.61, p=.063). The manipulations of working memory load or temporal task structure exerted no impact on any of the reaction time measures.

**Table 2:** Group Differences in Reaction Time and Error Measures.

	**MRT**	**MnRT**	**mu**	**RTSD**	**sigma**	**tau**	**CSD**	**om**	**corr**	**early**	**FB≤0.1**
**GROUP**	<1	<1	<1	15.6***	3.1+	18.1***	18.8***	9.7**	16.9***	16.8***	19.6***
**Controls**	634±118	630±118	546±103	142±40	108±36	79±33	181±46	3.2±3.4	83.7±7.5	5.7±5.4	5.6±0.5
**Patients**	652±127	635±129	531±130	186±41	125±33	127±48	240±53	7.7±7.5	72.3±12.0	1.2±1.8	6.2±0.4

Note: MRT: mean RT, MnRT: median RT; **µ**: mean of the Gaussian component of the RT distribution; RTSD: intra-subject standard deviation of reaction times; σ: standard deviation of the Gaussian component of the RT distribution; τ: mean and standard deviation of the exponential (ex-Gaussian) component of the reaction time distribution; con: consecutive standard deviation; om: percentage of omission errors; corr: percentage of correct responses; FB≤0.1: power in the pooled frequency bands of 0-0.1 Hz; GROUP: F-values of main effect

GROUP or indication of their non-significance (n.s.); ** p≤.01, *** p≤.001;

### 2: Frequency specificity of tau and the other measures of ISV

Figure **1c** clearly reveals that it is the lowest frequency band (0.001-0.025 Hz) that contributes most to τ. This frequency band corresponds to a cycle length of at least 40 seconds. Furthermore, the non-overlapping confidence intervals for the group means in τ after extracting the 0.001-0.025 Hz frequency band demonstrate the significantly greater impact of this slow frequency component on τ in patients compared to controls.

**Figure 2:**
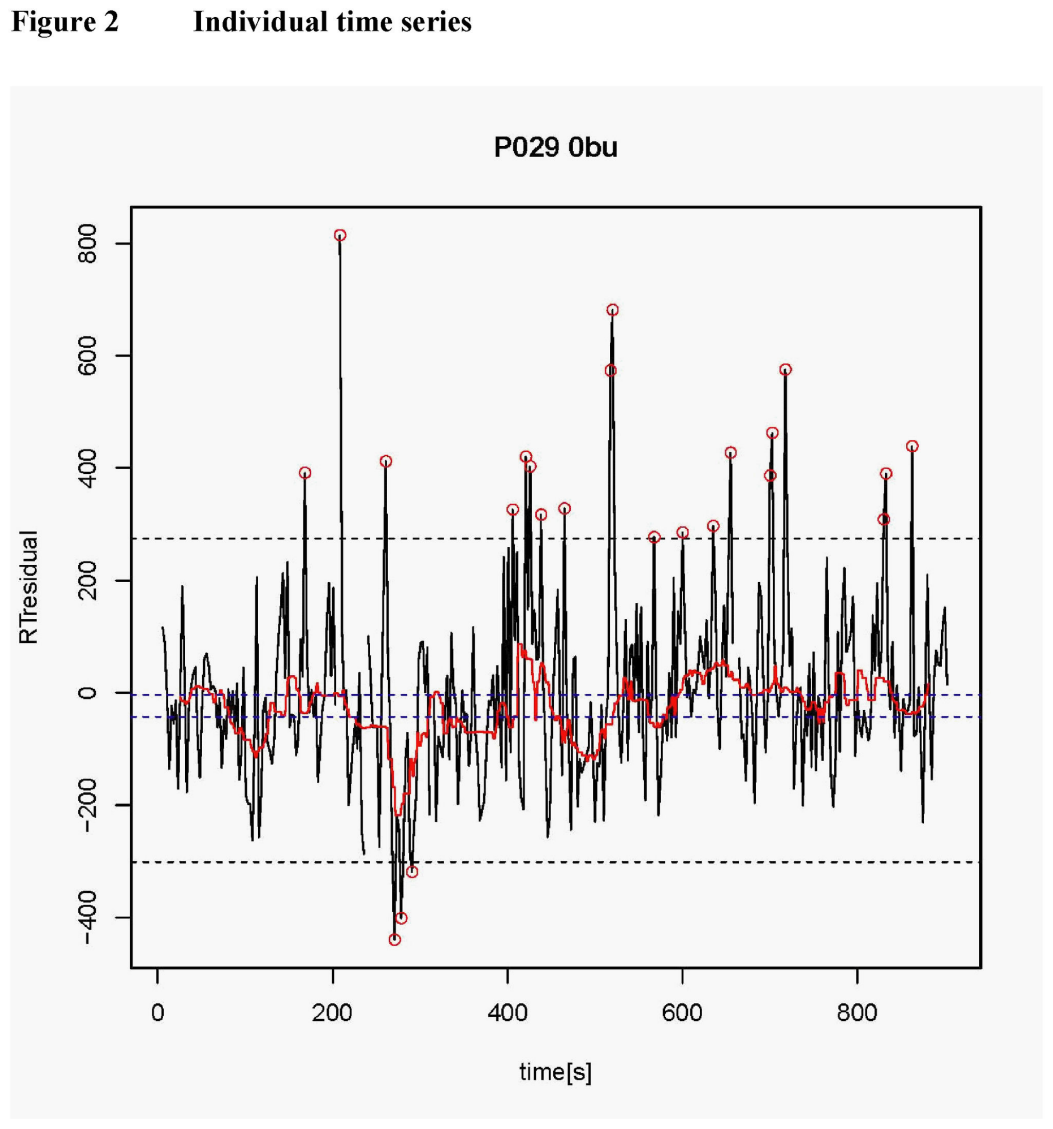
Typical residualized RT time series is shown for one patient (P029) and one condition (0-back non-jittered) for illustrative purposes. Successive RTs are connected with black lines; the outer horizontal dashed lines mark the threshold for particularly low and high RTs (Gaussian 1% threshold, cf. Methods). Very fast or slow reactions, exceeding the thresholds, are marked with circles. The red line shows the 40 s running median of values within the thresholds (background response fluctuation) and the inner horizontal dashed lines its tercile boundaries (1/3 of all values lying in each partition). The occurrence of each of these events (low/high value or omission) was associated with the tercile of the background response fluctuation. It can be seen that the few very fast RTs occur during a time of fast background RTs; similarly the larger number of very slow RTs (contributing to τ) occur preferentially during phases of slow background RT fluctuation.

Testing the association of particularly fast and slow RTs with background RT fluctuation (see Figure 2) revealed the following. In both patients (χ^2^=43.62, p<.0001) and control children (χ^2^=116.80, p<.0001), particularly slow responses occurred preferentially in the slow tercile of the background RT, whereas particularly fast responses occurred preferentially in its fast tercile (patients: χ^2^=35.25, p<.0001; controls: χ^2^=44.77, p<.0001). Conversely, omissions were evenly distributed across the background RT terciles both in patients (χ^2^=1.16, p=.56) and in controls (χ^2^=0.61, p=.74).

Regarding the other measures of ISV, RTSD and σ are less specific in their frequency composition than τ. Overall, RTSD values are larger than σ, and patients exhibit more variability across the entire frequency range than controls. Note that this RTSD analysis and the spectral power analysis (Figure **1b**) correspond mathematically. While RTSD shows significant group differences in the 0.001-0.025 Hz frequency band that characterises τ, σ does not.

The effect of RT time series filtering on CSD is shown in Figure **1d** for patients and controls. Numerical values, even for the full bands, are lower than for the original RT sequence (Table **2**) because the RT time series was interpolated to a sampling rate of 1 Hz for this analysis. It is obvious that CSD contains little (quasi-)periodicity and is non-specific to any frequency band. Still, this ISV facet is clearly more pronounced in patients than in controls. Overall the CSD spectrum is compatible with the fact that the square of CSD is the power of the temporal derivative of the RT time series: Low frequencies are suppressed and high frequency enhanced, relative to RTSD.

Group differences in the different variables are shown in Table **2**. Although the group difference in τ was strongest in the 0.001-0.025 Hz frequency band, the spectral power in the frequency range up to 0.1 Hz and RTSD differentiated patients and controls highly significantly (3.57≤t≤4.51), too. As the four RT power frequency bands below 0.1 Hz were positively correlated (controls: *r*≥.92; patients: *r*≥.78), we pooled them to the new frequency band variable “FB≤0.1”. Table **2** reveals that patients with ADHD showed significantly increased scores on all ISV measures except σ, with greatest group differences for τ, FB≤0.1 and CSD. Also, patients had more omissions than controls and gave fewer correct responses overall. While there was no significant GROUP difference for any measure of central tendency, patients were somewhat slower than controls regarding MRT, similar regarding MnRT and faster regarding **µ**.

Patients thus differed from controls in showing a greater exponential component (τ), more low-frequency spectral power and RT variance, overall and in consecutive trials, but normal Gaussian variability (σ) which, together, increased the arithmetic mean of the overall distribution despite somewhat *faster* Gaussian mean RT (µ).

## Discussion

The present study is the first systematic examination of the intra-individual relationships between measures of RT *dispersion* and RT *time series* in ADHD, focusing on the frequency specificity of τ, RTSD, σ, and CSD as a set of conceptually diverse and commonly used measures of ISV. Rather than determining inter-individual correlations between these variables [43], we developed a novel and more direct, two-level approach which, firstly, determines these relationships intra-individually and, secondly, outputs these individual data for group statistics.

The main new result of this study is that the group differences in the ex-Gaussian parameter τ are mostly driven by RT fluctuations below 0.025 Hz. This corresponds to a cycle length of at least 40 s. Additionally, we analysed blocks of 900s duration in which the RT time series was detrended, thereby eliminating systematic RT drifts and limiting the influence of very slow RT oscillations with a cycle length comparable to the block length or longer. This suggests that the particularly slow responses contributing to τ might occur with a quasi-periodic structure - rather than, for instance, occurring randomly across a block of trials or amassing just at the beginning or just the end of a task block. This interpretation was supported by a highly statistically significant clustering of particularly slow responses at the slow edges of a low-frequency background RT fluctuation (see Figure **2**). Leth-Steensen et al. [23] explained the “tau effect” in ADHD as a recurrence of lapses in attention due to deficient allocation of effort and thus cognitive-energetic factors [44]. This concurs with the interpretation of increased ISV in ADHD as “impaired state regulation” [17,8]. It is not in contradiction to the aforementioned interpretations to hypothesise that increased τ in ADHD with its low-frequency spectral signature might be related to “default mode” network abnormalities in ADHD [45]; This is all the more plausible as inefficient de-activation of “default mode” brain structures has been shown to be related with “lapses in attention” [24] or increased reaction time variability [46], at least in adults. Furthermore, it has been shown with electrocorticography that the default network is characterized by significant high gamma-band coherence, fluctuating at infra-slow (<0.1 Hz) frequencies and suggesting that the neurophysiological basis of DMN is quasi-periodic, infra-slow changes in local cortical activity [47]. The direct link established here between τ and slow frequency RT oscillations is thus in accordance with Sonuga-Barke & Castellanos’ [48] default mode interference (DMI) hypothesis (see also 49), according to which task performance in ADHD is modulated by endogenous fluctuations of attention with slow frequencies of less than 0.1 Hz. This is the spectral range of the four frequency bands that discriminated patients and controls best in the present study (see Table **1**). That this relationship holds for both groups, albeit much stronger for patients, suggests that DMI characterises children as young as those of the present study in general, but is increased in children with ADHD. The brain’s “default regions” seem to be intact and functioning at an early age [50], although their functional connectivity increases throughout childhood and adolescence [51].

The particularly slow responses appear to be embedded into the recurring slow phases of a large-scale background process that potentially reflects DMN activity. The specificity of this finding is underlined by the complete absence of a comparable accumulation of omissions in either tercile of the background RT fluctuation. Omissions, no less than particularly slow responses, can arguably be conceived of as “lapses in attention”. The temporal structure of their occurrence, however, indicates that they are not embedded in an on-going background RT fluctuation and thus reflect “lapses” with a different temporal structure; in this sense, particularly slow responses and omissions may be considered to reflect different kinds of “lapses”. This suggested dissociation concurs with a recent finding reported by Kuntsi and colleagues [15], who found omission errors and RT variability to load two different familial factors of cognitive impairment in ADHD.

Like τ, the other distributional measures of RT variability exhibit their own spectral signature. The frequency specificity of RTSD, for instance, appears to share the 1/f distribution characteristic of typical human RT data (for which logarithmic RT spectral power falls off quasi-linearly with increasing frequency; “1/f noise” [30,52] or the spectral content of spontaneous fluctuations of BOLD responses related to inter-trial variability of button press force [53]. It is thus tempting to speculate that the frequency spectrum of RTSD reflects the temporal dynamics of the coupling of certain functional brain systems with behaviour.

By contrast to both τ and RTSD, the parameter CSD extracts trial-to-trial differences in RT and therefore suppresses low frequencies and linearly emphasizes high frequencies. However, both power and group differences are maximum for low frequencies and minimum for high frequencies (see Figure **1b**). This has two implications. *Firstly*, the absence of group differences in the high frequencies as found in the present study, would argue against the notion that moment-to-moment fluctuations of attention are characteristic of ADHD [54]. Likewise, neuronal processes that would give rise to increases in such short-term fluctuations in attention (e.g., “neural noise” [55]) are not altered in ADHD according to the present data. *Secondly*, the absence of group differences in the high frequency fluctuations renders CSD primarily sensitive to group differences in the medium-to-low frequency range of approximately 0.05-0.10 Hz (10-20 sec cycle lengths; see Figure **1f**), despite its “computational” sensitivity to the high frequency fluctuations. This supports the above interpretation of our τ-related low-frequency variability increases and argues in favour of etiological models of ADHD that emphasise more general, regulatory processes [56,57]. Regulatory processes that are involved in generating time series with 1/f noise-like characteristics are presumably manifold, and include the combination of short-range processes with different characteristic time scales (such as the different human memory systems), strategy shifts or fluctuations in speed-accuracy criteria and the like [33]. Whether any of these processes is involved in generating the group differences in spectral power reported here remains to be determined.

## Conclusions and Potential Directions for Future Research

We have characterised distributional measures of ISV in terms of their spectral signature and thus linked two domains of ISV research that have been relatively unrelated so far, namely the *distributional* and *time series* approaches. Our results add to the ADHD literature by confirming the great sensitivity of the ex-Gaussian parameter τ to ADHD (see Tables **2** and **3**) and by showing for the first time that τ is “nourished” by slow quasi-periodic RT fluctuations that might be reflective of the default mode interference suggested to underlie ADHD psychopathology [48]. Obviously, proper neurophysiological data are required to substantiate this interpretation. Furthermore, we show that the “gold standard” ISV parameter, RTSD, picks up the “1/f noise” characteristic of RT fluctuations and that fast-frequency moment-to-moment fluctuations of performance do probably not contribute to the increases in ISV that are so characteristic of patients with ADHD.

**Table 3:** Overview of GROUP differences (F-values) in measures of ISV.

**Study**	**Age**	**MRT**	**SDRT**	**Mu**	**Sigma**	**Tau**	**CSD**	**Com**	**Om**
Leth-Stensen et al. 2000	10-11	22.9	50.1	1.4	0.2	**53.8**	―	―	―
Hervey et al. 2006	10	9.9	25.5	5.3	8.2	**28.7**	―	0.8	7.4
Vaurio et al. 2009	7-13	1.3	9.9^a^	0.1	4.5	6.9	―	**17.0**	
Kollins et al. 2009	31-32	―	―	**7.8**	n.s.	5.6	―	―	―
Buzy et al. 2009	7-14	―	―	0.10	6.2	**10.2**	―	1.2^b^	7.5

Note: ^a^ coefficient of variation (CV=SDRT/MRT) reported; ^b^ percentage accurate responses reported;

A potential limitation of our sample is its clinical heterogeneity. However, Rucklidge & Tannock [58] have found ISV to be the best predictor of both inattentive and hyperactive/impulsive symptoms.

Future ISV research should address the dimensionality of these possibly different facets of ISV and compare them, if possible, in the latent variable space, with respect to three key aspects of research on intra-subject variability in (neuro-) psychiatric disorders: reliability, nosological specificity including (differential) sensitivity to varying “contexts” (situations, tasks, internal states etc.; see also 3).
