# Correction: On the Temporal Characteristics of Performance Variability in Attention Deficit Hyperactivity Disorder (ADHD)

**DOI:** 10.1371/annotation/151a76cd-3bd7-457d-84eb-9b2e2fce3dff

**Published:** 2013-10-15

**Authors:** Bernd Feige, Monica Biscaldi, Christopher W. N. Saville, Christian Kluckert, Stephan Bender, Ulrich Ebner-Priemer, Klaus Hennighausen, Reinhold Rauh, Christian Fleischhaker, Christoph Klein

Two institutions that provided for payment of the publication charges for the article- a funding organization and university with which authors are affiliated that disbursed the funding- were incorrectly omitted from the funding statement. The Funding Statement should read: "The article processing charge was funded by the German Research Foundation (DFG) and the Albert Ludwigs University Freiburg in the funding programme Open Access Publishing." 

